# Association of immune-related adverse events with immune checkpoint inhibitor efficacy: real or imaginary?

**DOI:** 10.1186/s12916-020-01583-0

**Published:** 2020-04-21

**Authors:** Koji Haratani, Hidetoshi Hayashi, Kazuhiko Nakagawa

**Affiliations:** Department of Medical Oncology, Kindai University Faculty of Medicine, 377-2 Ohno-higashi, Osakasayama, Osaka 589-8511 Japan

**Keywords:** Immune-related adverse event, Immune checkpoint inhibitor, Immunotherapy, PD-1, PD-L1, Meta-analysis, Systematic review

## Background

Immune checkpoint inhibitors (ICIs) have become a standard of care for various types of cancer as a result of their demonstrated long-term survival benefit [1]. ICI therapy reinvigorates immune cells to achieve such treatment efficacy, but this mechanism of action can also give rise to autoimmune-like side effects known as immune-related adverse events (irAEs) [2]. The spectrum of irAEs is broad in that they can involve various organs, and, although they are usually mild or manageable, they can be life-threatening [2]. However, of note, evidence has suggested that these irAEs may be an early sign of ICI treatment success [3]. Indeed, numerous studies have revealed a positive association of irAEs with tumor response or extended survival in patients receiving ICI treatment. The fact that not all studies have confirmed such an association, however, has left the nature of the relation between irAEs and ICI treatment efficacy unclear [3].

In an article published recently in this journal, Zhou et al. approached this important clinical question by undertaking a comprehensive systematic review and well-designed meta-analysis [4].

## Systematic review identifies 30 articles for meta-analysis of PFS and OS

For their meta-analysis, Zhou et al. considered only studies that reported hazard ratios for progression-free survival (PFS) or overall survival (OS) in patients with irAEs compared with those without irAEs, resulting in the inclusion of 30 original articles for the analysis. These 30 articles consisted mostly of studies that evaluated patients with non-small cell lung cancer or melanoma who were treated with inhibitors of programmed cell death-1 (PD-1) or its ligand PD-L1, although patients with other cancer types or those treated with ipilimumab, an antibody to cytotoxic T lymphocyte antigen-4 (CTLA-4), were also included. The meta-analysis evaluated potential publication bias by funnel plot-based analyses with Egger’s test and the trim-and-fill method. It examined between-study heterogeneity by applying the *χ*^2^ test and *I*^2^ statistic, with a random-effects model being adopted when significant heterogeneity was observed, and sensitivity analyses were also performed.

## Positive association of irAEs with ICI treatment efficacy

Vigorous investigation of potential publication bias did not reveal serious bias in this study. The meta-analysis showed that both PFS and OS were significantly longer in patients with irAEs than in those without them. The hazard ratios were 0.52 and 0.54, respectively, meaning that the association was also clinically significant, although significant heterogeneity was detected by the *χ*^2^ test and *I*^2^ statistic (*p* < 0.01 and 50–75%, respectively). The authors evaluated the quality of each included original study with the Newcastle-Ottawa Scale [5], finding that quality was not clearly problematic (average and median scores of 5.47 and 5, respectively), even though most included studies were retrospective. One of the most important methodological issues in this type of study is how to alleviate lead-time bias, given that the exposure (irAEs) can fluctuate based on outcome (the better the treatment efficacy, the higher the frequency of irAEs). A landmark analysis is thus important to minimize this lead time bias [3, 6]. Of note, predefined sensitivity analysis including only studies that adopted landmark analysis also confirmed the positive association between irAE occurrence and better treatment outcome. In addition, predefined sensitivity analysis including only studies that performed multivariable analysis or those with a large sample size (*N* ≥ 100) did not alter the main conclusions of this meta-analysis.

A subgroup analysis according to irAE type or severity suggested that only cutaneous or endocrine irAEs as well as mild irAEs (grade 1 or 2) can be associated with better treatment outcomes. This was possibly due to the unnecessity for permanent discontinuation of ICI treatment [7] or for high-dose systemic glucocorticoid treatment, which can counteract antitumor immunity induced by ICI therapy [8]. These findings require a further clinical examination for verification, however. The subgroup analysis also did not reveal a difference among cancer types in the association of irAEs with treatment outcomes. With regard to ipilimumab treatment, such an association was not apparent, possibly as a result of the small number of studies included.

## This meta-analysis provides a limited generalizability

There is a possible concern with the application of the findings of this meta-analysis to clinical practice. Most of the included studies were retrospective observational studies, which may limit the quality of the analysis. Although meta-analyses in general provide a high level of evidence, they are dependent on the quality of the included studies. In particular, meta-analyses based on observational studies should be interpreted with caution. In this regard, the GRADE approach has been proposed as a means of evaluating the quality of meta-analyses. The quality of the present study can be estimated as low to moderate, given the possible risk of bias or inconsistency [9, 10], and its conclusions thus might not be perfectly generalizable for our clinical practice. However, given this type of investigation cannot be designed as an intervention study because irAEs are not experimental, a meta-analysis of observational studies may provide the best evidence possible.

## Future directions

Combination treatment with an ICI and other modalities including cytotoxic chemotherapy, molecularly targeted therapy, or localized therapy such as surgery or radiation is emerging as a new standard of care for many types of malignancies [1]. Whether the occurrence of irAEs during such combination therapy is associated with or predictive of long-term survival outcome is therefore of great clinical interest. Well-designed prospective observational studies that adopt both landmark analysis and multivariable analysis are needed to address this issue.

## Conclusions

The comprehensive systematic review and well-designed meta-analysis performed by Zhou et al. have provided important evidence for a positive association of irAE occurrence with a better outcome of ICI treatment—in particular, for the treatment of solid cancers with PD-1 or PD-L1 inhibitors.

## Data Availability

Not applicable.
